# Therapeutic Potential and Phytochemical Composition of *Santolina chamaecyparissus* From Kashmir Himalayas

**DOI:** 10.1155/sci5/7511508

**Published:** 2026-04-29

**Authors:** Mohmmad Ashaq Sofi, Mohd Abass Sofi, Anima Nanda, Aushiq Amin Ganaie, B. K. Nayak, Zulhabri Othman, Muhammad Zulfiqah Sadikan

**Affiliations:** ^1^ Department of Biomedical Engineering, Sathyabama Institute of Science & Technology, Chennai, 600119, Tamil Nadu, India; ^2^ Department of Chemistry, Sathyabama Institute of Science Technology, Chennai, 600119, Tamil Nadu, India; ^3^ Department of Education, Government of Jammu and Kashmir, Jammu and Kashmir, 192124, India; ^4^ Department of Botany, K. M. Government Institute for PG Studies and Research, Lawspet, Puducherry, 605008, India; ^5^ Department of Biochemistry, Faculty of Medicine, Manipal University College Malaysia, Melaka, 75150, Malaysia; ^6^ Department of Pharmacy and Health Sciences, University Kuala Lumpur Royal College of Medicine Perak, Ipoh Perak, 30450, Malaysia

**Keywords:** antimicrobial, antioxidant, cytotoxicity, GC–MS, medicinal plants, *Santolina chamaecyparissus*

## Abstract

Plant‐derived bioactive compounds are recognized as sustainable and promising alternatives to synthetic pharmaceuticals. This study examines the pharmacological efficacy of the methanolic leaf extract of *Santolina chamaecyparissus*, focusing on its antimicrobial, antioxidant, and anticancer properties. The extract was evaluated by well‐diffusion and broth microdilution against *Staphylococcus aureus*, *Enterococcus faecalis*, *Pseudomonas aeruginosa,* and *Escherichia coli,* as well as *Candida albicans*. MICs ranged from 64 μg/mL (*C. albicans*) to 256 μg/mL (*E. faecalis*), with 128 μg/mL for *S. aureus*, *E. coli*, and *P. aeruginosa.* The antioxidant potential was assessed using a DPPH radical scavenging assay, which revealed a dose‐dependent response with 59.23% inhibition at 100 μg/mL. Furthermore, the extract displayed potent cytotoxicity against A549 cells, with IC_50_ values of 85.38 μg/mL at 24 h and 72.46 μg/mL at 48 h, indicating its potential as an anticancer agent. Gas chromatography–mass spectrometry analysis identified key phytoconstituents potentially responsible for these bioactivities. These findings highlight the extract’s broad therapeutic promise, supporting *S. chamaecyparissus as* a candidate for further development in antimicrobial, antioxidant, and anticancer applications.

## 1. Introduction

Medicinal plants provide a rich and dependable source of bioactive molecules capable of promoting health and preventing disease [1]. Key constituents such as alkaloids, flavonoids, phenols, tannins, and lignans possess diverse therapeutic effects, including antioxidant, anticancer, antibacterial, and diuretic properties [2]. Consequently, ethnomedicinal studies are essential to develop innovative pharmaceutical compounds using herbal medicinal plants [3]. A significant proportion of synthetic drugs currently employed in treating human diseases are originally derived from plant sources. However, traditional therapeutic approaches face considerable limitations, primarily due to the serious adverse reactions associated with chemotherapeutic agents and the high cost of treatment. These limitations are particularly pressing in the context of cancer, which continues to be a major global health burden, with an estimated 9.9 million deaths reported in 2020 [4, 5]. In parallel, the growing issue of antimicrobial resistance among pathogenic bacteria has further compounded the healthcare burden. These concerns underscore the urgent need for the development of safer, more efficacious treatment options. [6]. Plants offer promising alternatives with the potential to treat a wide range of conditions, including infections and cancer, and provide antioxidant properties. Plant extracts are central in the quest for new drugs and represent valuable sources of phenolic and flavonoid compounds [7].

During the past few years, research has exploded into plant‐based antioxidants and their role in preventing diabetes, cancer, and cardiovascular diseases [8]. Both epidemiological and laboratory studies strongly indicate that phytochemicals with antioxidant properties hold promise in treating various ailments [9]. Compounds like phenols are already recognized for reducing the risk of degenerative diseases by alleviating oxidative stress and preventing macromolecular oxidation [10]. Therefore, developing innovative therapies with improved safety profiles is becoming a key focus in modern medicine [11]. Cancer, one of the most life‐threatening diseases today, is caused by both external and internal factors [12]. Despite the recent advancements in the development of new anticancer treatments, cancer remains a major global cause of mortality. Resistance to conventional chemotherapeutic agents poses a significant challenge in effectively managing cancer [13]. Therefore, exploring and developing innovative and potent anticancer agents to overcome this resistance has become paramount. Natural products have reemerged as a cornerstone of anticancer drug development, with contemporary pharmacopeias demonstrating that the majority of effective chemotherapeutic agents are either natural compounds or their synthetic analogs. Notably, around 60% of the drugs presently employed in cancer treatment originate from natural sources [14, 15]. Medicinal plants have now gained widespread acceptance as a viable alternative for cancer treatment in numerous countries globally [16].

*Santolina chamaecyparissus* L., a small perennial herb indigenous to the Mediterranean, parts of America, and Europe, is celebrated for its fragrant properties and has a longstanding role in traditional Mediterranean healing practices. It has many therapeutic benefits, including pain relief and antiseptic, anti‐inflammatory, antispasmodic, liver‐protective, antioxidant, and anticancer effects [17]. Although *S. chamaecyparissus* has been investigated for various medicinal properties in different regions, information on plants collected from the Kashmir Himalayan region remains limited. In this context, the present study examines the phytochemical composition and in vitro antimicrobial, antioxidant, and anticancer activities of *S. chamaecyparissus* from the Kashmir Himalayas. The findings provide region‐specific experimental evidence and help to improve understanding of the therapeutic potential of this species in relation to its unique geographic and environmental conditions.

## 2. Materials and Methods

### 2.1. Collection of Plant Material

**FIGURE 1:**
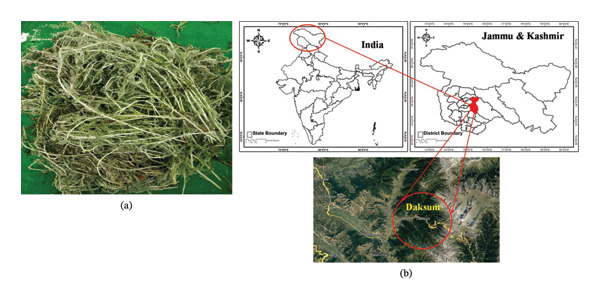
(a) *Santolina chamaecyparissus* plant (b) collection site Daksum, Anantnag, Kashmir.

Fresh leaf samples of *S. chamaecyparissus* were collected from Daksum village, located in the Anantnag District of Jammu and Kashmir. This site is situated at a latitude of 36.6°N and a longitude of 73.00°E, approximately 80 km from Srinagar, the capital of the state (Figure 1). Taxonomic authentication of the plant was conducted by experts at the University of Kashmir, where a voucher specimen (2735‐KASH) is preserved in the herbarium. Upon collection, the plant material was processed to remove dust particles by brushing off excess debris and rinsing it with distilled water. The washed plant material was then left to dry in the shade.

### 2.2. Extraction Process

Shade‐dried leaves of *S. chamaecyparissus* were finely powdered using a mechanical grinder. Twenty grams of the powdered leaf material was soaked in 200 mL of absolute methanol (99.9%) and placed on an orbital shaker at 23°C–25°C for 72 h. The filtrate was concentrated under reduced pressure using a rotary evaporator to obtain a crude methanolic extract, which was stored at 4°C until further analysis.

### 2.3. Phytochemical Analysis

The *S. chamaecyparissus* leaf extract was subjected to identify various phytochemical constituents (phenolic flavonoids, alkaloids, and terpenoids), using standard procedures [18].

### 2.4. GC–MS Analysis

Phytochemical analysis was performed utilizing the GC–MS Shimadzu‐QP2010 system. The sample was introduced into the capillary column via split injection (1:2), using helium (He) as the carrier gas at 1 mL/min. The analysis lasted for a total of 50 min. The protocol started at 80°C and gradually accelerated (3°C/min) to 200°C. A subsequent faster increase (10°C/min) reached 260°C, which was sustained for 5 min [19].

### 2.5. Microbes Used in the Study

Table​ 1 shows the list and collection of microbes used in the study.

**TABLE 1:** The list and collection of microbes used in the study.

Test organism	Source	Microbial type
*S. aureus*	ATCC 25923	Gram positive
*E. faecalis*	ATCC 29212	Gram positive
*E. coli*	ATCC 11229	Gram negative
*P. aeruginosa*	ATCC 15442	Gram negative
*C. albicans*	ATCC 10231	Fungus

### 2.6. Antimicrobial Assay

Microbial inocula (10^4^–10^6^ CFU/mL)were obtained from 18‐ to 24‐h cultures and plated onto nutrient agar under aseptic conditions. Six uniform wells, each 8 mm in diameter, were created in each agar plate. Plant extracts were prepared from a 10 mg/mL stock solution and carefully dispensed into the wells in volumes of 25, 50, 75, and 100 μL. Positive controls, including fluconazole, ampilox, and levofloxacin, were placed in individual wells. The plates containing the inoculum were then incubated at 37°C for 24 h, after which the inhibition zones were measured in millimeters.

## 3. Determination of the Minimum Inhibitory Concentration (MIC)

To determine the MIC, we used a 96‐well microplate assay adapted from Gabrielson et al. [20]. The first ten wells contained a dilution series of *S. chamaecyparissus* leaf extract, with concentrations ranging from 500 to 0.9 μg/mL. The test suspensions were standardized to a concentration of 1–5 × 10^6^ CFU/mL. The plates were then incubated at 37°C for 16 h. After incubation, 10 μL of MTT dye from a stock solution (5 mg/mL) was added to each well, and the plates were incubated for an additional 2 h at 37°C. Following this, 100 μL of dimethyl sulfoxide (DMSO) was added to each well as a solubilizer to facilitate visualization of the color change. A shift from yellow to purple indicated a positive result. The MIC was recorded as the lowest concentration of extract that completely inhibited microbial growth.

### 3.1. Antioxidant Activity of *S. chamaecyparissus*

To determine antioxidant activity, a DPPH assay was performed on the *S. chamaecyparissus* methanolic extract, using a lightly modified version of Brand‐Williams et al. [21]. The leaf extract of *S. chamaecyparissus* and ascorbic acid were used as standards in varying concentrations starting from 25 to 125 μg/mL. A solution of 1 mM of DPPH was prepared in methanol and subsequently was added in diluted concentrations of extracts and standard in sterile test tubes and shaken for some time in order to mix well and kept in dark conditions at room temperature for 30 min. In a similar manner, methanol and DPPH were used to prepare a blank solution without extract. Using the blank as a reference, the spectrophotometer measured the sample absorbance at 517 nm.

### 3.2. MTT Assay

The in vitro cytotoxicity of methanolic extract was carried out using the MTT assay as per previously used protocol with some modifications [22]. The human lung adenocarcinoma cell line (A549) was obtained from a certified cell repository (e.g., NCCS, Pune, India). Around 6 × 10^3^ A549 cells were seeded per well. The cells were exposed to a range of concentrations (12.5–100 μg/mL) of methanolic extracts of *S. chamaecyparissus and* then incubated for 24 h and 48 h with 5% CO_2_ at 37°C. Later on, 10 μL of MTT dye from the stock solution of (5 mg/mL) were added into each well and then again incubated for 4 h at 37°C. The cells treated with no extract were set as control. After the experiment, the media and MTT reagent were removed, and 100 μL of DMSO was added to each well, and the content was properly shaken. Finally, OD was measured at 590 nm, and the percentage of cell viability was determined using the following formula:

(1) Cell viability %=Absorbance of treated cellsAbsorbance of control cells×100.

### 3.3. Statistical Analysis

To determine statistical significance among the groups, we applied ANOVA and then used Tukey’s test to perform multiple comparisons.

## 4. Results

### 4.1. Phytochemical Analysis

**FIGURE 2:**
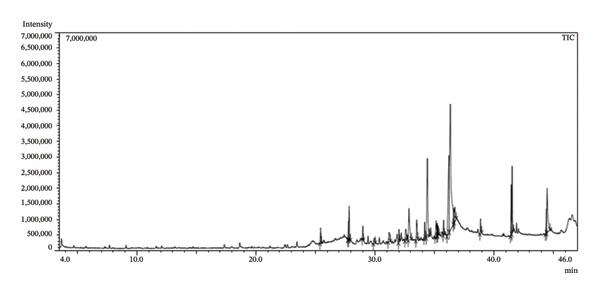
Chromatogram of the methanolic leaf extract from *S. chamaecyparissus*.

The phytochemical evaluation of the methanolic leaf extract of *S. chamaecyparissus* confirmed the presence of phenols, terpenoids, flavonoids, and alkaloids (Table 2). GC–MS analysis identified twenty‐five compounds, with oleic acid being the most prevalent. Other significant compounds included 9,12‐octadecadienoic acid (Z, Z), 4,8,13‐cyclotetradecatriene‐1,3‐diol,1,5,9‐trimethyl‐12‐(1‐methylethyl),2,3‐dihydroxypropylelaidate, 9‐octadecenoic acid, 1,2,3‐propanetriylester, and *n*‐hexadecenoic acid (Figure 2 Table 3).

**TABLE 2:** Qualitative phytochemical analysis of the *S. chamaecyparissus methanolic* extract.

Chemical test	Observation
Phenols	(+)
Alkaloids	(+)
Flavonoids	(+)
Terpenoids	(+)

*Note:* (+) means presence.

**TABLE 3:** The compounds detected through GC–MS analysis in the methanolic leaf extract of *S. chamaecyparissus*.

Peak#	Retention time	Area (%)	Compound name
1	25.439	1.22	Longiverbenone
2	27.785	1.55	3,7‐Octadiene‐2,6‐diol,2,6‐dimethyl‐
3	27.838	2.88	Oxalicacid,2‐isopropoxyphenyloctadecyle
4	28.984	1.12	3‐(2,2‐dimethyl‐6‐methylenecyclohexylidene)‐1‐methyl‐butyl acetate
5	29.933	0.81	(−)‐5‐Oxatricyclo [8.2.0.0 (4,6)] dodecane
6	31.190	1.53	Platambin
7	32.032	1.01	4,8,13‐Cyclotetradecatriene‐1,3‐DIOL
8	32.612	1.79	2 (1H)‐Naphthalenon,3,4,4a,5,6,7,8,8a. alpha‐octahydro‐5‐beta‐hydroxy‐4aalpha,7,7‐trimethyl‐, acetate
9	32.872	4.86	*n*‐Hexadecanoic acid
10	33.519	2.46	1‐Heptatriacotanol
11	34.189	1.24	2,4,5,5,8a‐Pentamethyl‐4a,5,6,7,8,8a
12	34.418	11.30	12‐Isopropyl‐1,5,9‐trimethyl‐4,8,13‐cyclotetradecatriene‐1,3‐diol
13	35.153	1.89	6‐Isopropenyl‐4,8a‐dimethyl‐1,2,3,5,6,7,8,8a
14	35.244	1.06	14b‐Pregnan
15	35.314	0.91	1,2‐Bis(allyloxy)‐4‐tert‐butylbenzene
16	35.772	1.27	Cyclotetradecane, 1,7,11‐trimethyl‐4‐(1‐methylethyl)‐
17	36.223	12.38	9,12‐Octadecadienoic acid (Z, Z)‐
18	36.349	28.23	Oleic acid
19	36.635	1.81	Longiborneol
20	36.723	1.94	Octadecanoic acid
21	38.879	1.10	Glycidol stearate
22	41.459	3.68	Ethyl (9z,12z)‐9,12‐octadecadienoate
23	41.543	5.50	9‐Octadecenoic acid,1,2,3‐propanetriylester
24	44.420	1.89	9,12‐Octadecadienoicacid (Z, Z)‐
25	44.484	6.59	2,3‐Dihydroxypropyl elaidate

		100.00	

### 4.2. Antimicrobial Activity

**FIGURE 3:**
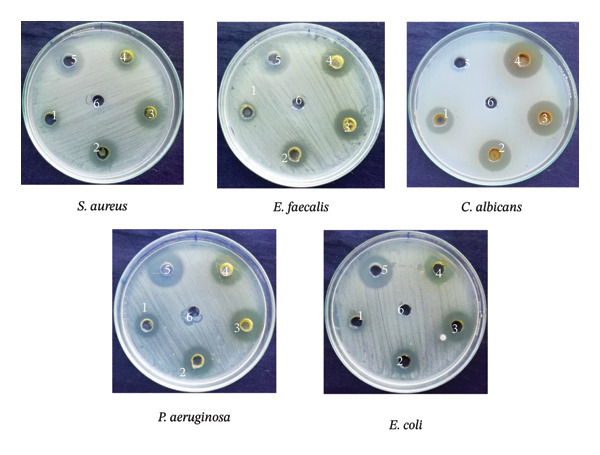
Antimicrobial activity of the methanolic leaf extract of *S*. *chamaecyparissus* against selected bacterial and fungal strains evaluated by the agar well diffusion method, demonstrating the antibacterial efficacy of methanolic leaf extract derived from *S. chamaecyparissus*.

**FIGURE 4:**
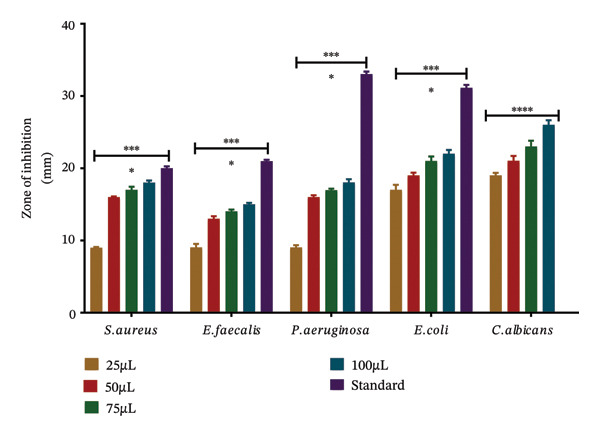
Antimicrobial efficacy of *S. chamaecyparissus* methanolic extract against different microbes, demonstrated by increasing zones of inhibition (mm) with increasing extract volumes (25–100 μL). SD represents standard drug (*n* = 3). We applied ANOVA and then used Tukey’s test to perform multiple comparisons.

In the agar well diffusion assay, increasing volumes (25, 50, 75, and 100 μL) of the methanolic leaf extract of *S. chamaecyparissus* were tested to evaluate dose‐dependent antibacterial activity. Wells were numbered on each Petri plate to represent extract concentrations (Wells 1–4), positive control (Well 5), and negative control (Well 6). Ampilox was used as a positive control for Gram‐positive bacteria, ciprofloxacin for Gram‐negative bacteria, and fluconazole for fungal strains, while DMSO served as the negative control*.* The methanolic leaf extract of *S. chamaecyparissus acted* against both bacterial groups and *Candida albicans* (Figures 3 and 4). The fungal target showed the greatest susceptibility in diffusion assays and the lowest MIC (64 μg/mL). Minimum inhibitory concentrations determined by broth microdilution were 64 μg/mL (*C. albicans*), 128 μg/mL (*Staphylococcus aureus, Escherichia coli, Pseudomonas aeruginosa)*, and 256 μg/mL (*Enterococcus faecalis),* supporting broad‐spectrum efficacy with enhanced antifungal potency.

### 4.3. Antioxidant Activity of *S. chamaecyparissus Leaf* Extract by DPPH Assay

**FIGURE 5:**
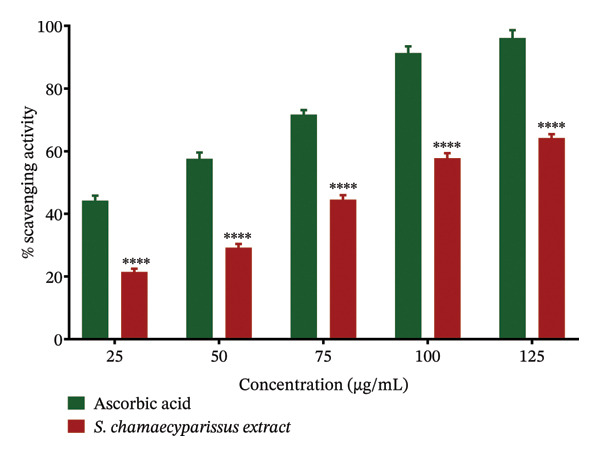
Comparative DPPH results for *S. chamaecyparissus* leaf extract and ascorbic acid. Values are shown as mean ± SD, and we applied ANOVA and then used Tukey’s test to perform multiple comparisons.

DPPH radical scavenging assay revealed that the crude leaf extract of *S. chamaecyparissus* possesses considerable antioxidant activity, although it was less potent than ascorbic acid at all tested concentrations (Figure 5). The extract and standard recorded IC_50_ values of 83.33 μg/mL and 35.50 μg mL^−1^, respectively, highlighting its potential as a plant‐derived antioxidant source worthy of further investigation.

### 4.4. Anticancer Activity

**FIGURE 6:**
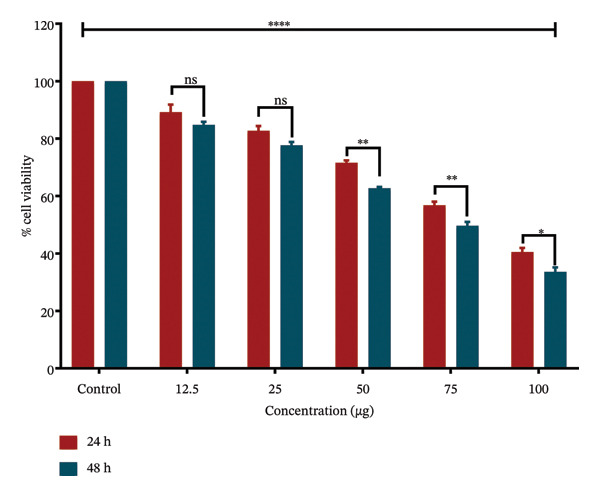
A549 cell viability after treatment with *S. chamaecyparissus* extract at various concentrations. Values are shown as mean ± SD, and we applied ANOVA and then used Tukey’s test to perform multiple comparisons.

**FIGURE 7:**
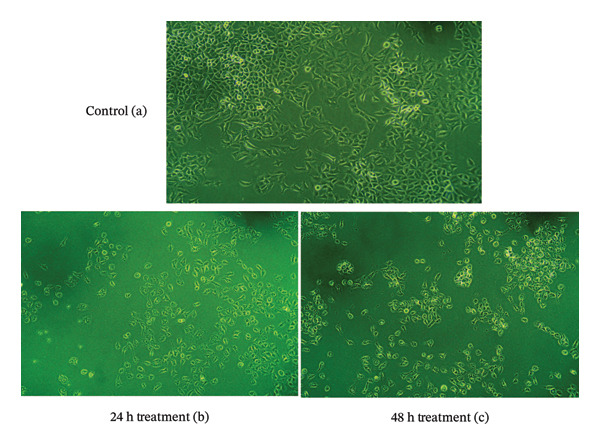
Morphological alterations in A549 cells following treatment with *S. chamaecyparissus* extract, observed using a phase‐contrast microscope. (a) Untreated control cells; (b) cells exposed to IC_50_ (85.38 μg/mL) for 24 h; (c) cells exposed to IC_50_ (72.46 μg/mL) for 48 h. Objective: 20 ×.

MTT assay results revealed that *S. chamaecyparissus* extract markedly reduced A549 cell viability in a time‐ and concentration‐dependent manner (Figures 6 and 7). The extract displayed IC_50_ values of 85.38 μg/mL at 24 h and 72.46 μg/mL at 48 h, indicating increased cytotoxic and antiproliferative potency over time. There was a noticeable decline in cell viability as the concentration of the extract increased, with the inhibitory effect becoming more pronounced after 48 h of treatment.

## 5. Discussion

GC–MS analysis of the methanolic extract of *S. chamaecyparissus* identified 25 phytocompounds with significant scientific, industrial, and biological importance. Oleic acid, the most prevalent identified compound in the leaf extract, has been shown to inhibit cell proliferation in diverse tumor cell lines by suppressing HER2 overexpression, a key oncogene in various human cancers. Furthermore, it affects intracellular calcium signaling pathways involved in cell proliferation. There is also evidence that oleic acid can trigger apoptosis in carcinoma cells, possibly by increasing ROS production or activating caspase 3 [23]. 4,8,13‐Cyclotetradecatriene‐1,3‐diol, 1,5,9‐trimethyl‐12‐(1‐methylethyl), has been reported to have antimicrobial action [24]. 9,12‐Octadecadienoic acid (Z, Z) exhibits anti‐inflammatory, cancer‐preventive, anti‐acne, hepatoprotective, and antimicrobial properties [25]. 2,3‐Dihydroxypropyl elaidate has been documented to possess antioxidant, anticancer, and antimicrobial activities [26]. *n*‐Hexadecenoic acid, found in *lime pomace*, demonstrated antibacterial, antioxidant, and antifungal properties alone or in combination [27]. Additionally, 2,3‐Dihydroxypropyl elaidate is an antispasmodic and immune modulator [28]. Ethyl (9z,12z)‐9,12‐Octadecadienoate exhibits hepatoprotective effects [29]. Platambin shows antibacterial effects [30]. A previous study revealed that 1‐heptatriacotanol displays antioxidant, anti‐inflammatory, anticancer, and antibacterial activities [31, 32]. Despite ongoing efforts by the pharmaceutical industry to develop new antibiotics, the proliferation of antibiotic‐resistant microorganisms presents a significant challenge for managing infectious diseases [33, 34]. The emergence of highly resistant microbial strains poses a considerable threat, especially to those with weakened immune systems. Natural plant‐derived compounds are being explored as promising solutions to combat this issue and to develop effective antimicrobial agents with low toxicity and broad‐spectrum activity. These compounds offer inspiration for the development of bioactive antimicrobial agents suitable for clinical use without requiring extensive chemical modifications [35]. People have become more interested in using plants as alternative medicines to treat infectious diseases in the past few years. These plants can serve as alternatives to synthetic drugs in various industries, reducing both their adverse effects on the body and manufacturing costs [36]. Recognizing the growing need for innovative therapies to address infections caused by antibiotic‐resistant microorganisms, Santolina plants offer promising opportunities as essential resources for developing these treatments. In this study, the methanolic extract demonstrated effectiveness against all studied microbes, with MIC values ranging from 64 to 256 μg mL^−1^. This indicates broad‐spectrum antibacterial activity, highlighting the potential of methanolic extracts to combat various microbial infections. Previous studies from different countries have shown the antimicrobial activity of this plant. Essential oil extracted from the Tunisian *S. chamaecyparissus flower* head showed potent antimicrobial activity against *P. aeruginosa* and *E. faecalis*. Similarly, the essential oil from *S. africana* displayed antimicrobial activity against various microorganisms, including standard strains such as *B. subtilis, E. faecalis, E. coli, P. aeruginosa, P. vulgaris, and S. aureus*, as well as the clinical strains: *Klebsella pneumoniae*, along with fungi like *A. flavus, A. niger*, and *C. albicans* [37]. The diameter of the inhibition zone was concentration‐dependent. Furthermore, the dichloromethane extracts from *S. chamaecyparissus* L. notably inhibited *Rhizopus stolonifera*[38]. The essential oil from Syrian *S. chamaecyparissus* leaves showed antibacterial and antifungal effects against *C. albicans*, *S. aureus*, *E. coli*, and *P. aeruginosa,* with MICs of 0.4–1.6 mg/mL [39]. Another study found that SEO was effective against *E. coli*, *B. subtilis, B. cereus, S. mutans,* and *C. albicans* (MICs of 0.5–2.0 mg/mL), with the lowest MIC of 0.5 μL/mL for *S. mutans* and *B. cereus* [40]. Our findings align with previous research. In our investigation, the methanolic extract exhibited efficacy against all tested microbes. The low MIC values (< 1 mg/mL) observed for the active extract of *S. chamaecyparissus* underscore its potent antimicrobial properties [41]. This finding is interesting, given the potential discovery of potent plant‐derived antimicrobial compounds. Compared with previous studies, the outcomes of the current investigation were notably superior, potentially due to differences in extraction methods and the presence of distinct phytocompounds in extracts from different plant species. The findings are encouraging, as the microbes in focus significantly contribute to illnesses acquired in community and healthcare settings. The increasing resistance of these microbes to the most potent available antibiotics underscores the critical need for new medications that target novel targets [42]. The strong antimicrobial potential of *S. chamaecyparissus is* evident from the MIC values recorded in this study, which were all considerably lower than 1 mg/mL. This finding supports the growing interest in plant extracts as reservoirs of novel antimicrobial agents. The underlying mechanisms of such activity may include damage to microbial membranes, inhibition of structural components such as cell walls, disruption of genetic material replication, or interference with enzymatic pathways. These actions collectively contribute to their effectiveness against various microorganisms, making plant extracts valuable sources of natural antimicrobial agents [43, 44]. Excessive oxidative compounds pose a threat to cellular integrity. To combat this, the body activates its antioxidant defense mechanisms to mitigate oxidative damage. However, oxidative stress may arise when this defense system is overwhelmed or insufficient [45]. Loss of oxidative balance is a significant pathological event, contributing to cellular damage and the onset of multiple chronic diseases, including neurodegenerative disorders, cardiovascular diseases, and cancer. In such circumstances, antioxidant supplementation may play a vital role in mitigating these adverse effects. Plant‐based antioxidants are gaining attention for their broad spectrum of bioactive compounds and health‐promoting effects [46]. In our study, the DPPH method was used to assess the antioxidant potential of the methanolic leaf extract of *S. chamaecyparissus*, with ascorbic acid as the reference. While ascorbic acid consistently outperformed the extract in terms of scavenging activity, the plant extract still showed considerable efficacy, achieving IC_50_ values of 83.33 μg mL^−1^ and 35.50 μg mL^−1^. The activity is probably attributable to phenols, flavonoids, and other redox‐active phytochemicals. The findings match earlier reports, underscoring the antioxidant capacity of different species within the same plant family and beyond. For instance, the essential oil of *Pluchea eupatorioides* exhibited significant antioxidant activity, with an IC_50_ value of 164.2 μg/mL [50], while the methanol extract of *Acmella oleracea* leaves recorded an IC_50_ of 198.34 μg/mL [48]. In *Vernonia amygdalina*, the ethyl acetate extract demonstrated the most substantial DPPH scavenging effect (IC_50_ = 658.28 μg/mL), followed by ethanol (636.01 μg mL^−1^) and aqueous extracts (340.22 μg/mL) [49]. Additionally, *Ageratum conyzoides* demonstrated a significant reduction in stable DPPH radicals with both its essential oil and methanolic extract, the latter displaying particularly potent antioxidant activity with an IC_50_ of 22.50 ± 3.18 μg/mL [50]. Similarly, our findings revealed that the methanolic extract of *S. chamaecyparissus* exhibited potent antioxidant activity, effectively neutralizing free radicals at a notably low concentration (IC_50_ = 83.3 μg/mL). By 2030, around 26 million new cancer cases will emerge, with an estimated 17 million deaths resulting from the disease [51]. Consequently, the demand for innovative, cost‐effective anticancer medications remains high. Lung cancer is the most fatal cancer, causing 18% of all cancer deaths in 2020. Traditional treatments such as radiation and chemotherapy do not work well for all lung cancer patients, highlighting the urgent need for new therapies that can be used in combination [52]. MTT assay analysis demonstrated that *S. chamaecyparissus* extract exerted significant cytotoxic effects on A549 cells, with cell viability decreasing proportionally to dose and exposure time. The IC_50_ values were calculated as 85.38 μg/mL at 24 h and 72.46 μg/mL at 48 h, indicating increased potency with prolonged exposure. These findings confirm the extract’s pronounced cytotoxic and antiproliferative effects, which intensified with higher concentrations and longer treatment durations. These observations align with previous studies showing the antilung cancer properties of extracts from plants in the same family as *S. chamaecyparissus.* Extracts from the *Anacyclus pyrethrum* plant demonstrated inhibition of lung cancer cell (A549) proliferation, with IC_50_ values of 225.3 and 156.6 for 24 and 48 h, respectively [53]. The ethanolic extract of *Centaurea solstitialis* flowers displayed differential cytotoxic effects across various cancer cell lines, with the lowest activity observed against A549 cells (IC_50_ = 252.5 μg/mL) [54]. The *n*‐hexane root extract of *Conyza canadensis* exhibited cytotoxic effects on A549 cancer cells (IC_50_ value of 94.73 μg/mL) [55]. Compared with previous studies, the outcomes of the current investigation were notably superior, potentially due to differences in extraction methods and the presence of distinct phytocompounds in extracts from different plant species. The results hold promise, as the targeted microbes are major contributors to hospital‐ and community‐acquired illnesses. The escalating resistance of these microbes to even the most potent antibiotics underscores the urgency for innovative medicines with novel targets.

The antibacterial, antioxidant, and cytotoxic properties of plant extracts are commonly associated with the chemical components present in the botanical mixture. Instead of relying solely on a single predominant active ingredient, the most effective use of a medicinal plant may result from the synergy of multiple components [56]. Several variables contribute to the wide range of chemical compositions among aromatic plant species and subspecies. These include local climatic conditions, seasonal variations, geographical location, geological influences, water availability, elevation above sea level, exposure to pests and fungal infections, the specific plant part used, and the extraction method [57]. The primary factor contributing to the differences in antibacterial and cytotoxic efficacy is the varying levels of secondary metabolites within the plant. Various factors, including differences in plant species and specific extraction methods employed, can influence these levels.

## 6. Conclusion

The methanolic leaf extract of *S. chamaecyparissus has shown* significant bioactivity in vitro, demonstrating strong antimicrobial, antioxidant, and anticancer effects, which underscores its pharmacological relevance. The extract exhibited potent efficacy against a broad range of pathogenic microbes, particularly against *C. albicans*. Its dose‐dependent antioxidant capacity and notable cytotoxicity against A549 lung cancer cells further reinforce its therapeutic value. GC–MS analysis provided valuable insights into the phytoconstituents likely responsible for these bioactivities. These results not only support the traditional medicinal applications of *S. chamaecyparissus* but also position it as a promising candidate for future drug development. Given the increasing interest in natural and sustainable alternatives to synthetic therapeutics, further research, including in vivo studies and the isolation of active compounds, is necessary to fully investigate its efficacy against infectious agents, disorders related to oxidative stress, and cancer.

**Nomenclature**
GC–MS: Gas chromatography–mass spectrometry
DPPH: (2,2‐Diphenyl‐1‐picrylhydrazyl)
SEO: *Santolina chamaecyparissus* essential oil

## Author Contributions

Mohmmad Ashaq Sofi drafted the manuscript, conducted the investigation, developed the methodology, performed data analysis, handled data curation, and contributed to resources, software, project administration, and study conceptualization. Mohd Abass Sofi drafted the manuscript review and editing and contributed to data interpretation and software analysis. Anima Nanda and B. K. Nayak contributed to drafting, supervised and validated the research, and participated in reviewing and editing the manuscript. Aushiq Amin Ganaie, Zulhabri Othman, and Muhammad Zulfiqah Sadikan assisted with writing, review and editing, data interpretation, and software analysis.

## Funding

No funding was received for this manuscript.

## Disclosure

All authors read and approved the final manuscript.

## Conflicts of Interest

The authors declare no conflicts of interest.

## Data Availability

All relevant data for this research are provided in the manuscript.
